# Effect of virtual reality-integrated mindfulness-based cognitive therapy on pain and psychological symptoms in chronic non-specific low back pain: protocol for a randomized controlled trial

**DOI:** 10.3389/fdgth.2026.1902063

**Published:** 2026-07-31

**Authors:** Panqi Wang, Junpeng Yuan, Qin Gu, Guoyang Dai, Xinran Zhu, Zhenxiu Liu, Youjia Yu, Xiaohong Jin

**Affiliations:** 1 Department of Pain Medicine, The First Affiliated Hospital of Soochow University, Suzhou, Jiangsu, China; 2 Department of Pain Medicine, Suzhou Xiangcheng People's Hospital, Suzhou, China; 3 Department of Pain Medicine, The Fourth Affiliated Hospital of Soochow University, Suzhou, Jiangsu, China; 4 Department of Anesthesiology, Sir Run Run Shaw Hospital, School of Medicine, Zhejiang University, Hangzhou, Zhejiang, China

**Keywords:** back pain bothersomeness, chronic non-specific low back pain, mindfulness-based cognitive therapy, oswestry disability index, virtual reality

## Abstract

**Background:**

Chronic non-specific low back pain (CNLBP) is a common condition associated with persistent pain, functional limitation, reduced quality of life, and substantial healthcare and socioeconomic burden. Mindfulness-based cognitive therapy (MBCT) has been increasingly used in chronic pain management and may provide a promising therapeutic approach for CNLBP. The emergence of virtual reality (VR) further provides an immersive, standardized, and accessible platform for delivering MBCT. This study aims to evaluate the effects and safety of a CNLBP-specific VR-integrated MBCT intervention in patients with CNLBP.

**Methods:**

This multi-center, prospective, parallel-group, open-label randomized controlled trial will recruit 214 patients with CNLBP from the pain clinics of three hospitals. Participants will be randomly assigned in a 1:1 ratio to either an 8-week VR-MBCT group consisting of weekly in-clinic VR sessions supplemented by structured audio-guided home practice, or a usual care control group receiving usual care alone, such as pharmacotherapy and physical therapy, using stratified block randomization according to study center and age (≥60 years or <60 years), with block sizes of 2 and 4. The primary outcomes are the proportions of patients achieving at least a 30% improvement from baseline in functional disability, assessed by the Oswestry Disability Index (ODI), and in self-reported back pain bothersomeness, assessed on a 0–10 scale, at week 8. Secondary outcomes include pain intensity, anxiety, depression, sleep quality, kinesiophobia, pain catastrophizing, work productivity, system usability, and use of concomitant treatments. Safety outcomes will include VR-related adverse events, such as vertigo, vomiting and nausea, headache and falls. Assessments will be conducted at baseline, week 4, week 8, and week 26. Data will be analyzed according to the intention-to-treat principle using generalized estimating equations (GEEs).

**Discussion:**

This study aims to provide preliminary evidence regarding the efficacy and safety of VR-integrated MBCT as an adjunct to usual care in patients with CNLBP.

**Clinical Trial Registration:**

https://clinicaltrials.gov/ct2/show/NCT07589790, identifier NCT07589790.

## 1 Introduction

Chronic non-specific low back pain (CNLBP) is a common musculoskeletal condition associated with persistent pain, functional limitation, reduced quality of life, and substantial healthcare and socioeconomic burden (1–4). Unlike low back pain caused by specific spinal pathology, CNLBP lacks a clearly identifiable structural cause (5). Conventional treatments, including pharmacotherapy and physical therapy, often focus on peripheral nociception and physical impairment, but may insufficiently address the cognitive, emotional, and behavioral dimensions of pain (6, 7). This may partly explain why many patients continue to experience persistent symptoms and functional disability despite treatment (8). Moreover, abnormal imaging findings do not necessarily correlate with pain severity (9, 10), suggesting that CNLBP can not be fully explained or managed through structural or pharmacological approaches alone (11).

Modern pain science increasingly emphasizes the biopsychosocial model, in which biological, psychological, and social factors jointly contribute to the development and persistence of chronic pain (12). Psychological factors such as pain catastrophizing, fear-avoidance beliefs, anxiety, and depression are closely involved in the maintenance of CNLBP (12–14). Accordingly, psychological interventions, including cognitive behavioral therapy (CBT) and mindfulness-based stress reduction (MBSR), have been used in chronic pain management and have shown beneficial effects in reducing pain and improving related outcomes (15–17). However, some patients obtain limited benefit from these approaches (18, 19), and their implementation may be constrained by therapist availability, time requirements, and patient adherence (20).

Mindfulness-based cognitive therapy (MBCT) integrates elements of CBT and mindfulness-based approaches, helping patients relate differently to pain-related thoughts, emotions, and bodily sensations through awareness and acceptance (21). Evidence for mindfulness-based interventions in chronic pain is promising but not conclusive. Recent reviews suggest potential benefits for pain and psychological symptoms, although the certainty of evidence varies across outcomes and is limited by small samples, intervention heterogeneity, and risk of bias (19, 22). Evidence specific to MBCT is more limited, with a meta-analysis reporting improvements in depressive symptoms but no clear effects on pain intensity or pain interference (23). Nevertheless, emerging studies suggest potential benefits for functional outcomes in chronic pain conditions (24–27). However, conventional MBCT usually requires experienced therapists and repeated face-to-face sessions, which may limit its accessibility in routine clinical settings (27).

Virtual reality (VR) has increasingly been used to deliver psychological and behavioral interventions for chronic back pain. In patients with chronic low back pain, home-based VR programs combining pain neuroscience education, cognitive-behavioral strategies, breathing, relaxation, and brief mindfulness exercises have improved pain intensity and pain interference (28). VR neuroscience-based therapy incorporating body awareness, attentional and emotional training, and pain reappraisal has also shown beneficial effects in patients with chronic back pain (29). A recent meta-analysis of 20 randomized trials found that VR-based interventions improved pain, pain-related fear, and disability in chronic low back pain (30). However, the authors of the meta-analysis noted that benefits driven by distraction within the virtual environment may diminish once patients return to daily life, highlighting the need for transferable psychological skills beyond the VR setting. Moreover, most existing VR-based pain interventions—including the FDA-authorized RelieVRx™—have been designed around a multi-modal “skills kit” model that integrates mindfulness exercises within broader cognitive-behavioral or relaxation frameworks rather than grounding the entire curriculum in a structured, MBCT-driven therapeutic protocol (31). While these approaches have demonstrated short-term efficacy, they do not systematically train patients to fundamentally transform their relationship with pain through sustained, progressive practice.

Integrating VR with MBCT may therefore provide a scalable platform for delivering these principles through immersive experiential learning, where abstract concepts such as bodily awareness and cognitive defusion can be concretized in interactive virtual environments. A recent study reported the feasibility and acceptability of VR-MBCT in patients with depression (32). However, VR-MBCT protocols specifically designed for chronic non-specific low back pain (CNLBP) remain underdeveloped, and high-quality randomized controlled evidence regarding their efficacy and safety is still lacking. Our present intervention addresses this gap: the content and progression of the entire 8-week program are based on MBCT, with weekly sessions progressing from attentional grounding to recognizing habitual reactions, decentering, and practicing mindful movement adapted for CNLBP. In this protocol, VR serves as an integrated delivery platform that guides and reinforces these specific MBCT practices rather than functioning primarily as a distraction tool or a repository of isolated coping techniques.

This study aims to evaluate the efficacy and safety of a CNLBP-specific VR-MBCT intervention for patients with CNLBP. We hypothesize that, compared with usual care, an 8-week VR-MBCT program will result in greater improvement in functional disability and pain bothersomeness.

## 2 Methods

This study protocol was approved by the Ethics Committee of the First Affiliated Hospital of Soochow University (Approval No. 2026306, Approval Date: March 19, 2026). Written informed consent will be obtained from all participants prior to enrollment. All study procedures will be conducted in accordance with the Declaration of Helsinki and Good Clinical Practice (GCP) guidelines. Additionally, this protocol was developed in accordance with the SPIRIT (Standard Protocol Items: Recommendations for Interventional Trials) guidelines, as outlined in Supplementary File S1.

File 1. SPIRIT 2013 Checklist: Recommended items to address in a clinical trial protocol and related documents.

### 2.1 Study design and patients

**Figure 1:**
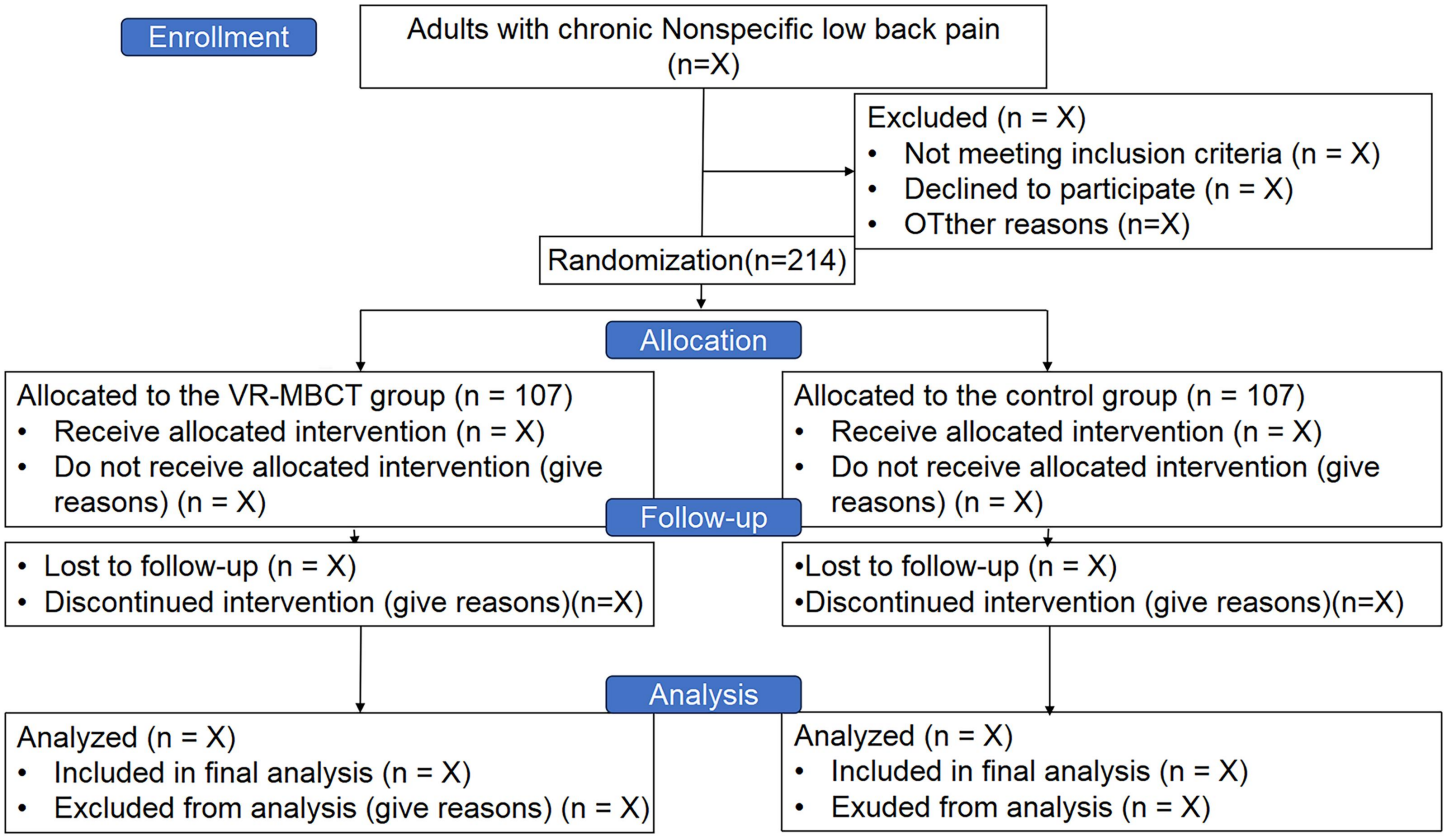
Study ﬂow diagram showing the enrollment and randomization process.

This study is a multi-center, prospective, randomized, parallel-group, open-label clinical trial designed to evaluate the efficacy and safety of VR-integrated MBCT in patients with CNLBP. The trial will be conducted at the First Affiliated Hospital of Soochow University, Suzhou Xiangcheng People's Hospital, and The Fourth Affiliated Hospital of Soochow University, enrolling a total of 214 patients. Recruitment and follow-up are scheduled to take place between May 2026 and January 2027. Data collection is expected to be completed by February 2027, and data analysis by March 2027. Study flow diagram showing the enrollment and randomization process is shown in Figure 1.

### 2.2 Inclusion criteria

Patients who meet the following criteria will be included:

- Diagnosed with CNLBP according to standard criteria (3), with pain duration ≥3 months.
- Aged 20–70 years (inclusive).
- Average self-reported back pain bothersomeness ≥4(scale, 0–10) in the past week.
- Provided informed consent and family members will be informed of the study as support persons.

### 2.3 Exclusion criteria

The exclusion criteria include:

- Coexisting pain conditions that may interfere with the assessment of CNLBP.
- Severe neurological or psychiatric disorders (e.g., unstable epilepsy, psychosis, dementia).
- Severe psychiatric comorbidities (e.g., active suicidal ideation, severe depression requiring antidepressant medication).
- History of substance abuse (including alcohol or drugs) in the past year.
- History of accidents or injuries related to back pain in the past year.
- Severe visual impairment, vertigo, or history of VR-induced motion sickness precluding safe VR use.
- Inability to communicate effectively to express subjective feelings.

### 2.4 Primary outcome

The primary outcome is defined as the proportions of patients achieving at least a 30% improvement from baseline in functional disability and self-reported back pain bothersomeness at week 8. Functional disability will be assessed using the Oswestry Disability Index (ODI), and back pain bothersomeness will be measured on a 0–10 scale (0 indicates not at all bothersome; 10 indicates extremely bothersome. The ODI is a validated instrument for assessing disability in patients with chronic low back pain, with or without radiculopathy (33). It consists of 10 items scored on a 6-point Likert scale, with total scores ranging from 0 to 100%, where higher scores indicate greater disability (34). Back pain bothersomeness reflects the overall subjective burden of pain and is consistent with the multidimensional perspective of the biopsychosocial model. A treatment responder is defined as a patient achieving at least a 30% improvement from baseline at the primary 8-week endpoint. This responder definition was selected based on the international consensus proposed by Ostelo et al., which recommended a 30% improvement from baseline as a practical threshold for identifying clinically meaningful change in individual patients (35). This threshold has subsequently been widely adopted in low back pain research (36, 37) and has demonstrated validity in predicting patient satisfaction in large real-world validation studies (38). The co-primary composite endpoint requires concurrent improvement in both disability and pain bothersomeness, providing a stringent and clinically interpretable definition of treatment response.

### 2.5 Secondary outcome

Secondary outcomes will be assessed at baseline (T0), week 8 (T2), and week 26 (T3), unless otherwise specified. Pain intensity will be measured using the Numerical Rating Scale (NRS), ranging from 0 to 10, with higher scores indicating greater pain intensity. Depressive symptoms will be assessed using the Patient Health Questionnaire-9 (PHQ-9), and anxiety symptoms using the Generalized Anxiety Disorder-7 (GAD-7), with higher scores indicating more severe symptoms. Physical activity will be evaluated using the International Physical Activity Questionnaire–Short Form (IPAQ-SF). Sleep quality will be assessed using Athens Insomnia Scale, with higher scores indicating poorer sleep quality. Fear-avoidance beliefs related to physical activity will be measured using the Fear-Avoidance Beliefs Questionnaire–Physical Activity subscale (FABQ-PA), and pain catastrophizing will be assessed using the Pain Catastrophizing Scale (PCS), with higher scores indicating greater fear-avoidance beliefs and pain catastrophizing, respectively. Work productivity and activity impairment will be evaluated using the Work Productivity and Activity Impairment questionnaire (WPAI), with higher percentages indicating greater impairment. Global improvement will be assessed at weeks 8 and 26 using a patient-reported global improvement measure, with higher scores indicating greater perceived improvement. Use of any treatments, including pharmacological and non-pharmacological therapies, will be recorded at baseline, week 8, and week 26. System usability will be assessed in the intervention group only using the System Usability Scale (SUS) at week 8, with higher scores indicating better usability. Patient satisfaction will be assessed in both groups at weeks 4, 8 and 26.

### 2.6 Safety outcomes

Adverse events will be monitored at each participant contact. VR-related symptoms, including dizziness, nausea and vomiting, headache, eye soreness, and falls, will be recorded. These symptoms are expected to be mostly mild and transient. If a participant experiences a fall or significant discomfort, the intervention session will be paused or discontinued as appropriate. Serious adverse events will be reported to the Data and Safety Monitoring Committee within 24 h.

### 2.7 Randomization and blinding

Randomization will be performed using a computer-generated sequence with a 1:1 allocation ratio. Stratified block randomization will be applied according to study center and age (≥60 years or <60 years), with block sizes of 2 and 4. Allocation concealment will be ensured using sequentially numbered, sealed, opaque envelopes. Due to the nature of the intervention, participants and VR therapists can not be blinded. However, outcome assessors and statisticians will remain blinded to group allocation throughout the study.

### 2.8 Study interventions

The VR-MBCT program was specifically developed for patients with chronic non-specific low back pain. All participants followed the same standardized 8-week MBCT sequence. Condition-specific adaptations included VR scenarios addressing pain-related bodily sensations, thoughts, emotions, and movement avoidance; mindful movement combined with low-back-friendly rehabilitation exercises; and real-time feedback on posture and movement amplitude. Exercise difficulty could be adjusted within prespecified limits according to performance, whereas the therapeutic content and progression remained standardized.

The intervention will be delivered through a VR-MR system co-developed by the research team and Hangzhou Yijian Technology Co., Ltd. The system consists of a PICO Ultra VR-MR headset, which provides immersive mixed-reality experiences, and a DM-TG01A motion capture camera, which enables real-time tracking of participants' movements and postures, as well as feedback and correction through the VR headset. All equipment will be provided to participants free of charge.

Participants assigned to the intervention group will attend one facilitator-guided, in-clinic VR-MBCT session per week for 8 weeks, with each session lasting approximately 30 min. Between sessions, they will complete audio-guided mindfulness practice at home three times per week, with each practice lasting 20–30 min. No VR equipment will be used for home practice. Each in-clinic session will be automatically generated by the system and facilitated by trained research staff, who will assist with equipment setup, safety monitoring, and procedural guidance. The 8-week progressive curriculum is summarized in Supplementary Table S1 and includes the following themes: automatic pilot and awareness, including body scan and mindful breathing; body scan and movement repertoire, including cat-cow exercises and visual feedback; mindful movement and graded exposure, including system-adjusted spinal and core exercises; responding rather than reacting, including the ABC model and urge surfing; cognitive defusion, including thought replacement exercises; self-compassion, including acceptance narratives and posture transitions; relapse prevention and self-care, including compassionate letter writing and loving-kindness meditation; and maintenance planning and real-world transfer, including system- guided training and individualized maintenance planning. A detailed breakdown of weekly themes, VR core scenes, and key teaching points is provided in Supplementary Table S1.

System-generated data will include training duration, module completion, and pause events. Self-reported data, including ABC practice logs and scene preferences, as well as anxiety scores assessed using a 0–10 visual analogue scale before and after each VR session, will be collected through the system interface. Research staff will conduct weekly telephone follow-ups to promote participant adherence. If a participant misses a VR session, a brief motivational interview will be conducted to identify barriers and encourage continued participation.

Participants in the control group will receive standard clinical care, including pharmacological treatment, such as analgesics, and physical therapy, such as extracorporeal shock wave therapy. They will not receive structured VR training during the initial 8-week study period. After completing the 6-month follow-up assessment, participants in the control group may receive the same 8-week VR-MBCT program according to their preferences. Group allocation will remain concealed from outcome assessors and statisticians until study completion.

Randomization will be conducted before the study begins by an independent researcher using a computer-generated randomization sequence with stratified block randomization according to study center and age (≥60 years or <60 years), using block sizes of 2 and 4. Group assignments will be concealed using sequentially numbered, sealed, opaque envelopes, which will only be opened after the participant is enrolled and assigned. Due to the nature of the VR-based intervention, blinding of participants and intervention therapists is not feasible. However, outcome assessors and statisticians will remain blinded to group allocation throughout the study to minimize detection bias. The schedule for patient enrollment, study interventions, and outcome assessments will adhere to the SPIRIT statement (Supplementary Table S2).

### 2.9 Data collection and monitoring

Patient information will be collected from electronic medical records and baseline self-report questionnaires. Variables will include age, sex, body mass index, education level, employment status, pain duration, comorbidities, current treatment use, expectations for pain improvement, and other relevant clinical characteristics. Significant life events, such as major illness, accidents, bereavement, or changes in employment status, will also be recorded. As all participants will be recruited from outpatient clinics, hospital admission will not be required. Follow-up data will be collected through in-person visits at weeks 4 and 8 and telephone interviews at week 26, and then entered into a secure electronic data capture system.

To ensure data accuracy and integrity, all data will be independently double-entered by two trained research coordinators. Discrepancies will be resolved through discussion or consultation with the principal investigator. Regular monitoring and audits will be conducted by an independent data quality supervisor to verify source documents against entered data. Research staff involved in data collection will receive standardized training in case report form completion and protocol adherence before trial initiation. A dedicated Data Monitoring Committee will oversee data quality, Good Clinical Practice compliance, and protocol deviations.

Serious adverse events, regardless of their relationship to the VR intervention, including dizziness, headache, falls, syncope, nausea, or vomiting, will be reported promptly to the principal investigator. Appropriate measures will be taken to ensure participant safety. Serious adverse events will also be reported to the Data Monitoring Committee within 24 h for assessment and, if necessary, modification or discontinuation of the study protocol.

### 2.10 Sample size calculation

The sample size was calculated based on the co-primary outcomes: response rates for functional disability and self-reported back pain bothersomeness. The primary outcome is defined as the proportion of patients achieving clinically meaningful improvement, defined as at least a 30% improvement from baseline, in both functional disability and back pain bothersomeness after 8 weeks of intervention. Based on previous studies of mind-body interventions for chronic low back pain (15, 39), we assumed that 35% of participants in the usual care group and 55% of participants in the VR-MBCT group would achieve clinically meaningful improvement, corresponding to an absolute between-group difference of 20 percentage points. With a two-sided significance level of 0.05, 80% power, and a 1:1 allocation ratio, 97 patients per group were required for each co-primary outcome. This sample size was considered sufficient to provide adequate power for both co-primary outcomes; the study will be considered positive only if both outcomes reach statistical significance. Allowing for a 10% dropout rate, the total sample size was set at 214 patients, with 107 patients in each group. Sample size calculations were performed using PASS software, version 15.0 (NCSS, LLC, USA).

### 2.11 Statistical analysis

All analyses will follow a prespecified statistical analysis plan and will be conducted according to the intention-to-treat principle, whereby participants will be analyzed according to their randomized group assignment regardless of intervention adherence. Continuous variables will be summarized as means with standard deviations or medians with interquartile ranges, as appropriate, while categorical variables will be summarized as frequencies and percentages. All statistical tests will be two-sided, and statistical significance will be defined as a *P* value <0.05.

For the co-primary outcomes, separate regression models will be fitted for functional disability and self-reported back pain bothersomeness. Outcomes from all follow-up time points (weeks 4, 8, and 26) will be analyzed simultaneously using generalized estimating equations (GEEs) to account for within-participant correlations over time. Because treatment effects may vary across follow-up periods, models will include fixed effects for treatment group, time point, and treatment-by-time interaction terms. Baseline values of the corresponding outcome measures, study center, age group (≥60 years vs. <60 years), sex, pain duration, and other clinically relevant baseline covariates associated with outcomes or missingness will be included as adjustment variables.

The co-primary outcomes are binary responder outcomes, defined as at least a 30% improvement from baseline. Therefore, modified Poisson regression models with a log-link function and robust sandwich variance estimators will be used within the GEE framework to estimate relative risks and corresponding 95% confidence intervals. The primary endpoint will be evaluated at week 8. The study will be considered positive only if both co-primary outcomes reach statistical significance at the primary endpoint.

Continuous secondary outcomes, including pain intensity, psychological scores, sleep quality, physical activity, fear-avoidance beliefs, pain catastrophizing, and work productivity, will be analyzed using linear GEE models or linear mixed-effects models, as appropriate according to outcome distributions and covariance structures. Secondary outcomes assessed only at selected time points will be analyzed using models including the corresponding follow-up assessments.

Missing outcome data will initially be handled under a missing-at-random assumption using likelihood-based estimation within the GEE framework and adjustment for predictors of missingness. Sensitivity analyses will be performed using multiple imputation methods and per-protocol analyses to evaluate the robustness of the findings under different missing-data assumptions.

Safety analyses will include all participants who received at least one intervention session. Adverse events will be summarized descriptively and compared between groups using chi-square tests or Fisher exact tests, as appropriate.

All analyses will be performed using R software (R Foundation for Statistical Computing, Vienna, Austria) or SPSS software (IBM Corp., Armonk, NY, USA).

### 2.12 Patient and public involvement

Neither patients nor members of the public were involved in the design, conduct, recruitment, or reporting of this study. Study results will be shared with participants via email upon study completion.

## 3 Discussion

This multi-center, prospective, randomized, parallel-group, open-label clinical trial involves patients with chronic non-specific low back pain and aims to evaluate the efficacy and safety of VR-integrated mindfulness-based cognitive therapy as an adjunctive intervention. The intervention combines weekly facilitator-guided, in-clinic VR-MBCT sessions with structured audio-guided mindfulness practice at home, allowing participants to reinforce the skills introduced during the VR sessions in their daily lives. The primary objective is to determine whether an 8-week VR-MBCT program improves treatment response, defined as at least a 30% improvement from baseline in both functional disability and self-reported back pain bothersomeness at week 8. Secondary objectives include evaluating changes in pain intensity, psychological symptoms, sleep quality, physical activity, fear-avoidance beliefs, pain catastrophizing, work productivity, use of concomitant treatments, system usability, patient satisfaction, and safety outcomes, including dizziness, headache, nausea, vomiting and falls. This study will be conducted and reported in accordance with the SPIRIT guidelines.

This study seeks to overcome some limitations of conventional therapy by integrating VR with MBCT. VR technology can provide a highly standardized and immersive training environment, thereby ensuring consistency across intervention sessions. In addition, visualization and gamified elements may help patients better understand and practice relatively abstract MBCT concepts. This immersive and interactive format may support engagement by making practices such as bodily awareness and decentering more concrete. Attendance at the weekly in-clinic sessions and completion of prescribed home-based audio practice will be recorded to evaluate adherence to the present intervention. This approach may support engagement, in contrast to traditional models that rely heavily on therapist expertise and patient self-motivation (32, 40).

The role of MBCT in chronic pain management has been supported by emerging evidence. In a randomized controlled trial conducted by Melissa Day and colleagues involving 69 patients with chronic low back pain, participants were assigned to MBCT, cognitive behavioral therapy (CBT), or mindfulness meditation (MM). The MBCT group demonstrated high treatment expectancy and satisfaction. Intention-to-treat analyses showed that MBCT was superior to MM in improving pain intensity and depressive symptoms, while achieving effects comparable to CBT in improving physical function. Importantly, these benefits were maintained at 6-month follow-up with medium-to-large effect sizes and were observed even in patients with comorbid depression or long disease duration (mean duration 14.46 years) (24). These findings suggest that MBCT, as an integrative intervention, may simultaneously target pain perception, emotional regulation, and functional recovery. However, conventional MBCT relies heavily on face-to-face therapist guidance and requires sustained patient attention and motivation. Patients with chronic low back pain often exhibit poor treatment adherence, with reported adherence rates ranging from 50% to 70% (41), which may limit the broader implementation of MBCT in routine clinical practice.

The integration of VR and MBCT may operate through several complementary mechanisms. First, VR provides a safe and controllable environment for graded exposure, which may reduce fear-avoidance beliefs and facilitate relearning that spinal movement is safe (42). Second, immersive VR may support the experiential delivery of MBCT by enhancing presence, embodied interaction, and engagement during practices involving bodily awareness, decentering, acceptance, and mindful movement (43, 44). These features may allow participants to engage with the intervention from a first-person perspective rather than solely through passive observation. Third, standardized VR delivery may improve treatment consistency and reduce therapist-related variability. Finally, the 8-week hybrid intervention model, combining weekly in-clinic VR sessions with home-based audio practice, is designed to balance treatment intensity with practical accessibility. This structure, supplemented by automated adherence reminders, may support sustained engagement throughout the program (45).

It is also worth distinguishing our VR-MBCT intervention from existing VR-based behavioral programs for chronic pain, most notably the FDA-authorized RelieVRx™ (46). While RelieVRx™ delivers a fully self-administered, in-home multi-skills training, our intervention is organized around a complete, facilitator-guided MBCT curriculum that integrates mindful movement with low back-specific rehabilitation exercises. Rather than positioning VR-MBCT as a replacement for existing approaches, we view it as offering an alternative therapeutic pathway for patients whose pain is accompanied by prominent cognitive and emotional components.

The week-26 follow-up was selected as a clinically relevant time point to evaluate the maintenance of treatment effects, as this duration has been commonly used in trials of mind-body interventions for chronic low back pain (15, 28), allowing comparison with existing literature while balancing resource requirements. A 6-month follow-up period provides a clinically meaningful window to assess whether the skills cultivated through VR-MBCT—particularly acceptance, decentering, and mindful movement—translate into sustained functional and psychological improvements. Moreover, this time frame facilitates direct comparison with existing studies of MBSR, CBT, and VR-based interventions, while balancing the practical challenges of longer follow-up periods, such as participant attrition.

However, several limitations of this study should be acknowledged. First, due to the nature of the intervention, participants can not be blinded to group allocation, which may introduce performance and reporting bias. Nevertheless, outcome assessors and statisticians will remain blinded throughout the study, and the primary outcomes are based on validated patient-reported instruments. Second, the usual care control design can not fully account for non-specific effects, including attention and expectancy effects. However, participants in the control group will receive the same intervention after completion of follow-up, which may help address ethical considerations. Third, the 26-week follow-up period may be insufficient to evaluate long-term maintenance effects. Future studies should include longer follow-up periods and direct comparisons with conventional face-to-face MBCT interventions.

In conclusion, this study will provide preliminary evidence regarding VR-MBCT as a standardized and scalable non-pharmacological adjunctive intervention for CNLBP. If proven effective, VR-MBCT may have substantial potential for broader implementation in pain management settings, thereby helping reduce the burden of chronic low back pain on patients and healthcare systems.

## Data Availability

Data collection is ongoing therefore data and materials are not available. As such, there are currently neither publications containing the results of this study already published nor manuscripts in which results are described submitted to any journal.
