# Supplementary material for: Effect of virtual reality-integrated mindfulness-based cognitive therapy on pain and psychological symptoms in chronic non-specific low back pain: protocol for a randomized controlled trial
Source: Front Digit Health. 2026 Jul 31;8:1902063. doi: 10.3389/fdgth.2026.1902063 (PMC13482564; doi:10.3389/fdgth.2026.1902063)
Supplement: Supplementary file 2 [file Table1.pdf]

S1 Table 1. Progressive 8-week VR-MBCT curriculum

Week	Theme	VR Core Scene	Key Teaching Points and Interactions
1	Automatic Pilot and Awareness	Forest and lake	• PNE: Introduction to pain neurophysiology and sensitization mechanisms<br>• Body scan (MR mode)<br>• Formal practice: mindful breathing/lying<br>• Automatic pilot vs. mindful awareness comparison
2	Body Scan and Movement Repertoire	Mirror-based virtual space	• Guided body scan: directing attention to body regions with visual feedback<br>• Cat-cow spinal movements in VR<br>• Movement exploration and comfort zone expansion
3	Mindful Movement and Graded Exposure	Moving platform / Bridge / Cloud	• Graded exposure training: combining body scan, cat-cow, spinal rotation, standing, forward flexion, single-leg balance, shoulder bridge, and core activation<br>• System-generated difficulty adjustment based on performance
4	Responding vs. Reacting: Pain and Stress	Valley and river (floating boat)	• Interoceptive awareness: mountain color changes (high altitude = pain alert; valley = pain relief)<br>• ABC model: Event (A) → Belief (B) → Consequence (C)<br>• Urge surfing: observing body sensations being "washed" by water
5	Cognitive Defusion: Thoughts Are Not Facts	Mind laboratory	• ABC learning: selecting real-life events (A), extracting distressing beliefs (B), observing consequences (C) with color coding<br>• Thought replacement experiments: swapping distressing cards for positive (green) cards<br>• Mindful movement: wall-supported standing, mini-squats, heel raises
6	Self-Compassion: Stopping the Pain Battle	Dry riverbed / Posture transition	• VR narrative: character stops fighting, expands breathing<br>• ABC exercise: replacing "must fight" with acceptance<br>• Posture transition: after drying, riverbed becomes vast open field
7	Relapse Prevention and Self-Care	Warm and safe space (fireplace, blanket, dim lighting)	• Compassionate letter writing in VR<br>• Self-soothing and self-compassion practices<br>• ABC relapse prevention: using self-care to replace distressing events, observing C changes<br>• Loving-kindness meditation
8	Maintenance Planning and Real-World Transfer	Daily life scenes (kitchen, office, park)	• System-guided training: identifying and managing daily stressors, selecting coping tools (breathing space, ABC replacement, mindful movement,

Week	Theme	VR Core Scene	Key Teaching Points and Interactions
			loving-kindness)<br>• Dry riverbed revisit: comparing with Week 1<br>• Integrated loving-kindness meditation (15 – 20 minutes)<br>• Personalized maintenance plan (predictive signals, coping strategies)
