# Supplementary material for: Effect of virtual reality-integrated mindfulness-based cognitive therapy on pain and psychological symptoms in chronic non-specific low back pain: protocol for a randomized controlled trial
Source: Front Digit Health. 2026 Jul 31;8:1902063. doi: 10.3389/fdgth.2026.1902063 (PMC13482564; doi:10.3389/fdgth.2026.1902063)
Supplement: Supplementary file 3 [file Table2.pdf]

S1 Table 2. SPIRIT schedule of patient enrolment, study interventions and outcome assessment.

Time point	Enrolment	Allocation	Post-allocation	Follow-up		
	clinic visits	Pre-intervention	Week 1	Week4	Week 8	Week 26
Patient enrolment						
Eligibility criteria	×					
Written informed consent	×					
Demographic data	×					
Baseline characteristics	×					
Randomization		×				
Allocation		×				
Study interventions						
VR-MBCT			←	—	→	
Usual care			←	—	→	
Outcome assessment						
ODI	×			×	×	×
Back pain bothersomeness	×			×	×	×
Pain intensity	×				×	×
PHQ-9	×				×	×
GAD-7	×				×	×
IPAQ-SF	×				×	×
AIS	×				×	×
FABQ-PA	×				×	×

PCS	×				×	×
Global improvement					×	×
WPAI	×				×	×
Concomitant treatments	×				×	×
SUS					×[1]	
Patient satisfaction					×	
Safety outcomes						
Vertigo			←	—	→	
Nausea and vomiting			←	—	→	
Headache			←	—	→	
Falls			←	—	→	

[1] Only assessed in the VR-MBCT intervention group, not collected in the control group. VR-MBCT, Virtual Reality- based Mindfulness- Based Cognitive Therapy; ODI, OswestryDisability Index; PHQ- 9, Patient Health Questionnaire- 9; GAD- 7, Generalized Anxiety Disorder- 7; IPAQ- SF, International Physical Activity Questionnaire- Short Form; SQS, Sleep Quality Scale; FABQ- PA, Fear- Avoidance Beliefs Questionnaire- Physical Activity subscale; PCS, Pain Catastrophizing Scale; WPAI, Work Productivity and Activity Impairment questionnaire; SUS, System Usability Scale; AE, Adverse Event; VR- related AEs include vertigo, nausea and vomiting, headache, and falls; SPIRIT, Standard Protocol Items: Recommendations for Interventional Trials.
